# Identification of Novel Piperazinylquinoxaline Derivatives as Potent Phosphoinositide 3-Kinase (PI3K) Inhibitors

**DOI:** 10.1371/journal.pone.0043171

**Published:** 2012-08-14

**Authors:** Peng Wu, Yi Su, Xianghong Guan, Xiaowen Liu, Jiankang Zhang, Xiaowu Dong, Wenhai Huang, Yongzhou Hu

**Affiliations:** 1 Zhejiang University - École Normale Supérieure Joint Laboratory of Medicinal Chemistry, College of Pharmaceutical Sciences, Zhejiang University, Hangzhou, Zhejiang, China; 2 Institute of Pharmacology and Toxicology, College of Pharmaceutical Sciences, Zhejiang University, Hangzhou, Zhejiang, China; Univ of Bradford, United Kingdom

## Abstract

**Background:**

Development of small-molecule inhibitors targeting phosphoinositide 3-kinase (PI3K) has been an appealing strategy for the treatment of various types of cancers.

**Methodology/Principal Finding:**

Our approach was to perform structural modification and optimization based on previously identified morpholinoquinoxaline derivative WR1 and piperidinylquinoxaline derivative WR23 with a total of forty-five novel piperazinylquinoxaline derivatives synthesized. Most target compounds showed low micromolar to nanomolar antiproliferative potency against five human cancer cell lines using MTT method. Selected compounds showed potent PI3Kα inhibitory activity in a competitive fluorescent polarization assay, such as compound **22** (IC_50_ 40 nM) and **41** (IC_50_: 24 nM), which induced apoptosis in PC3 cells. Molecular docking analysis was performed to explore possible binding modes between target compounds and PI3K.

**Conclusions/Significance:**

The identified novel piperazinylquinoxaline derivatives that showed potent PI3Kα inhibitory activity and cellular antiproliferative potency may be promising agents for potential applications in cancer treatment.

## Introduction

The phosphoinositide 3-kinase (PI3K) family includes lipid kinases that catalyze the phosphorylation of the 3′-hydroxyl group of phosphatidylinositols to generate second messengers, such as phosphatidylinositol-3,4,5- triphosphate (PIP_3_) [1], [2]. PIP_3_ recruits downstream effectors along the PI3K/protein kinase B (PKB orAkt)/mammalian target of rapamycin (mTOR) signaling cascade that is of crucial importance for the regulation of cellular growth, survival, and proliferation [3]. Based on sequence homology and substrate preference, PI3Ks are divided into three classes. Class I PI3Ks are subdivided into four isoforms, PI3Kα, PI3Kβ, PI3Kδ, and PI3Kγ, according to different activation mechanism and varied catalytic and regulatory subunits [4]. Many studies have demonstrated that gain-of-function mutations in the gene encoding the catalytic subunit of PI3Kα, *PIK3CA*, amplification of *PIK3CA*, and loss-of-function mutations in PTEN, a lipid phosphatase that dephosphorylates PIP_3_ result in constitutive activation of the PI3K signaling cascade, which contributes to tumor growth and progression [5], [6], [7]. These observations make targeting PI3Ks, especially PI3Kα, with small-molecule inhibitors a promising strategy for cancer therapy [8], [9], [10], [11].

Considerable efforts have been devoted toward the development of small-molecule inhibitors targeting PI3K with more than twenty promising molecules have been progressed into various stages of clinical trials [11], [12].

**Figure 1:**
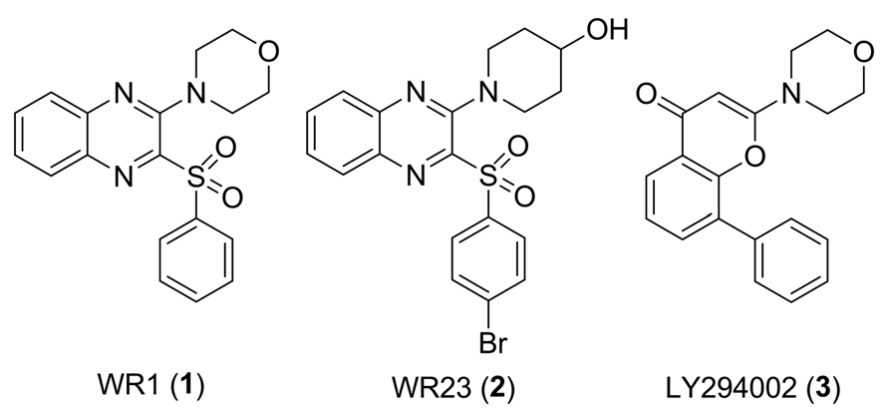
Morpholinoquinoxaline WR1, piperidinylquinoxaline WR23, and LY294002.

In our efforts to identify novel inhibitors of PI3K [13], we established a pharmacophore model based on reported PI3K inhibitors and identified the morpholinoquinoxaline derivative WR1 (**1**) as an initial hit with good potency against PI3Kα (IC_50_: 0.44 µM) [14], which is equivalent to that of the extensively studied tool compound LY294002 (**2**, PI3Kα, IC_50_: 0.63 µM) (Fig. 1) [15], [16]. Following modification based on WR1 led to the discovery of a series of piperidinylquinoxaline derivatives with good to potent PI3Kα inhibitory activity and cellular antiproliferative activity, such as WR23 (**3**, PI3Kα, IC_50_: 0.025 µM) (Fig. 1) [17]. In this paper, we describe our ongoing efforts in this field that led to the identification of this series of novel piperazinylquinoxaline derivatives as potent PI3Kα inhibitors.

**Figure 2:**
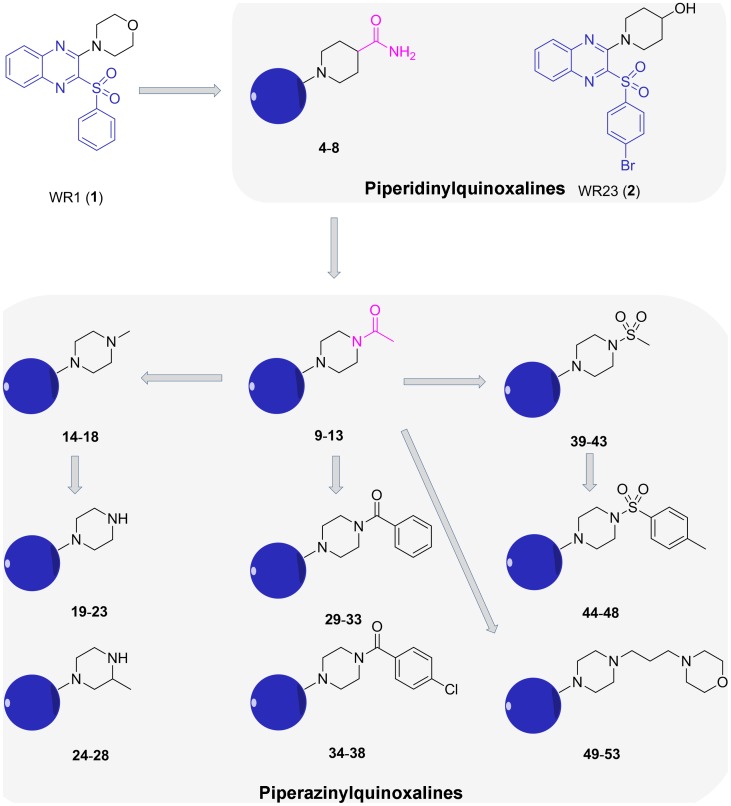
The modification and optimization journey from WR1 to target piperazinylquinixaline derivatives. Blue circles of compounds 4–53 stand for an arylsulfonylquinoxaline moiety.

Among compounds synthesized based on modifying the 4-morpholino group at the 2-position of the quinoxaline scaffold of WR1, compounds **4–8** with a 4-carbamoylpiperidin-1-yl group at the 2-position of the quinoxaline were identified as interesting leads for further study due to their potent *in vitro* antiproliferative activity that was equivalent to that of WR23. Thus, compounds 4–8 were chosen for further optimization. Reversion of the carboxamide group at the 4-position of the piperidinyl ring of **4–8** led to compounds **9–13** with a 4-acetylpiperazin-1-yl group. To fully assess the impact of different piperidinyl substituents on cellular and enzymatic potency, modification in the following facets were made. Firstly, replacement of the 4-acetyl group on the piperazinyl ring with a smaller group, i.e. methyl, led to compounds **14–18**. Removing the 4-methyl group and relocating the 4-methyl group as 3-methyl group on the piperazinyl ring led to compounds **19–23** and **24–28**, respectively. Secondly, replacement of the 4-acetyl group of **9–13** with a benzoyl or 4-chlorobenzoyl group afforded compounds **29–33** and **34–38**, respectively, with a larger substituted piperazinyl group than that of **9–13**. Thirdly, replacement of the 4-acetyl group of **9–13** with a methylsulfonyl or 4-methylphenylsulfonyl group led to compounds **39–43** and **44–48**, respectively. Lastly, different from above rigid substituted piperazinyl group, a flexible 4-(3-morpholinopropyl)piperazin-1-yl group was introduced to the 2-position of the quinoxaline scaffold to afford compounds **49–53** (Fig. 2). This work led to the identification of a series of piperazinylquinoxaline derivatives, whose synthesis, *in vitro* evaluation, apoptosis inductive effort, and docking analysis are described herein.

## Results and Discussion

### Chemical Synthesis

**Figure 3:**
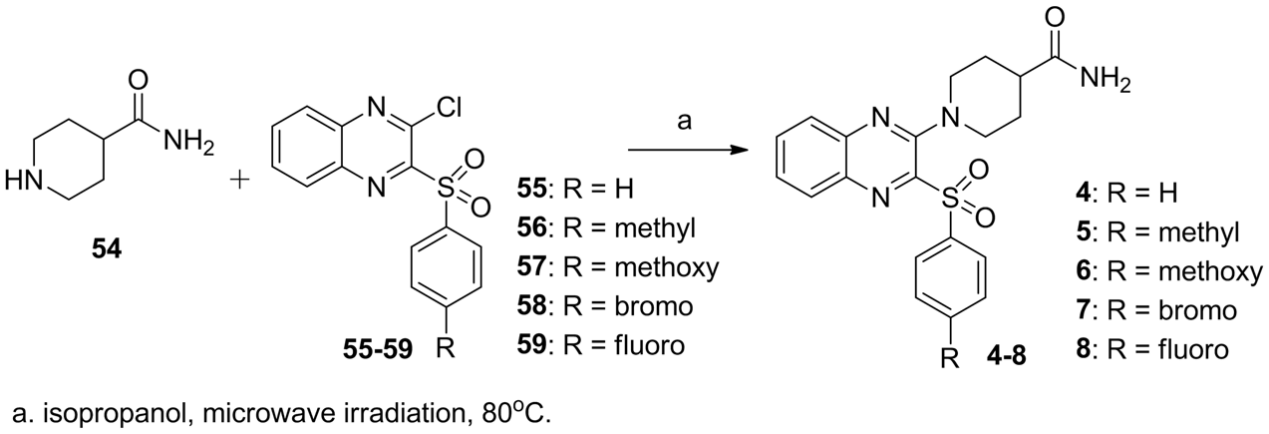
Synthesis of piperidinylquinoxalines 4–8.

As shown in Figure 3, piperidinylquinoxalines **4–8** were obtained by a microwave-assisted reaction of *N*-carbamoylpiperazine **54** with 2-chloro-3-arylsulfonylquinoxalines **55–59**. 2-Chloro-3-arylsulfonylquinoxalines **55–59** were synthesized using the same materials and procedures as reported [13].

**Figure 4:**
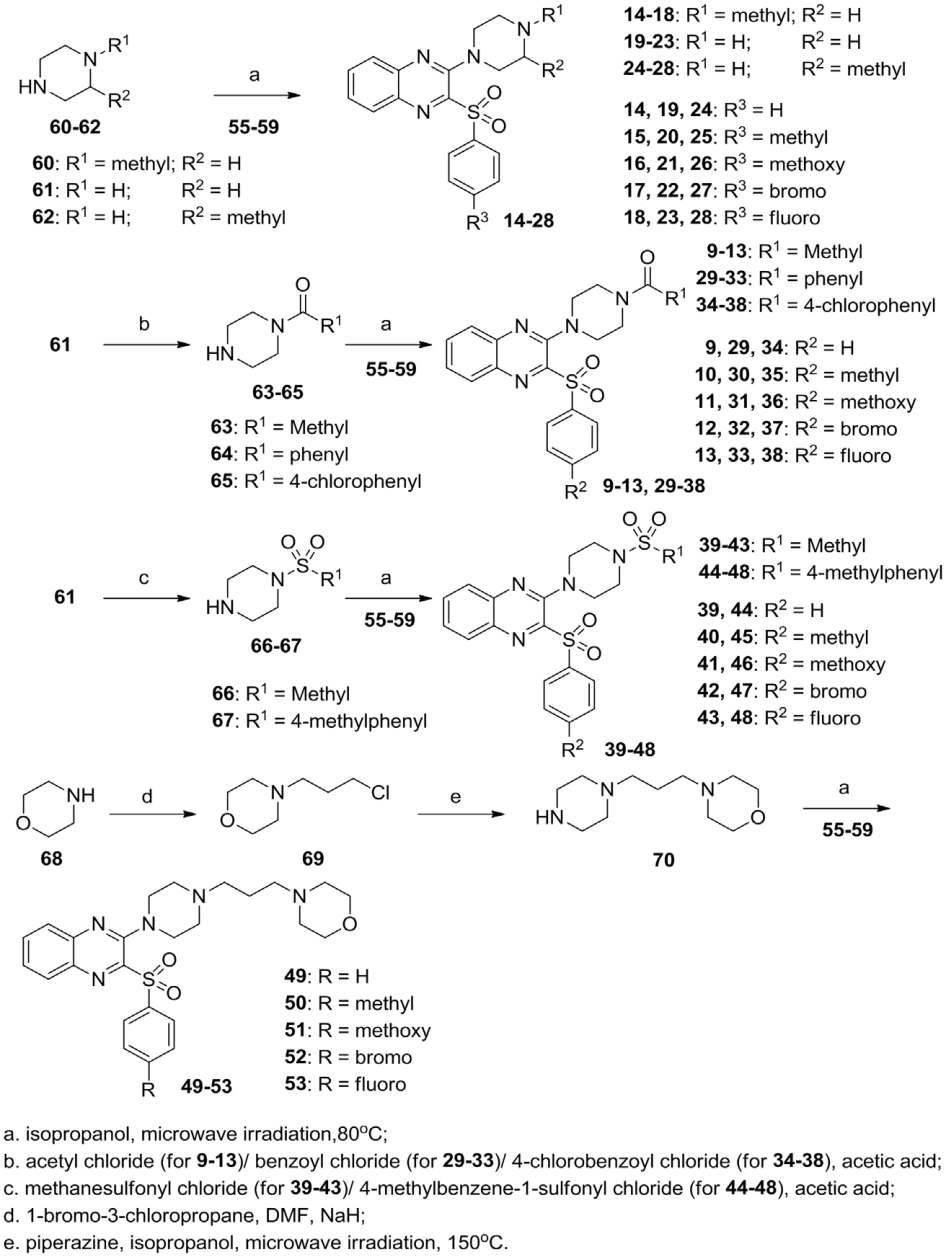
Synthesis of piperazinylquinoxalines 9–53.

As shown in Figure 4, for the synthesis of piperazinylquinoxalines **9–53**, similar materials and procedures were applied as synthesis of compounds **4–8** except for the use of compounds **60–67** and **70** instead of *N*-carbamoylpiperazine. Intermediates **63–67** were prepared using reported procedure [18], [19]. *N*-3-(morpholinopropyl)piperazine (**70**) was prepared by a reaction of piperazine with 4-(3-chloropropyl)morpholine (**69**), which was obtained by a reaction of morpholine with 1-bromo-3-chloropropane [20].

Fifty new derivatives including forty-five piperazinylquinoxalines were synthesized. Their purities were above 95% indicated by HPLC.

### Biological Evaluation and Structure-Activity Relationships (SAR)

#### Antiproliferative activity against human cancer cell lines

All synthesized target compounds were firstly tested for their antiproliferative activity against five human cancer cell lines, PC3, A549, HCT116, HL60, and KB, using MTT assay. Compounds WR1 and LY294002 were used as positive controls. As shown in Table 1, 2, 3, both pieridinylquinoxalines **4–8** and piperazinylquinoxalines **9–53** exhibited significantly improved antiproliferative activity against most tested cell lines than that of WR1 and LY294002, for example, compounds **4–8** showed IC_50_ ranging from 1.17 to 4.36 µM against PC3 cell, compounds **14–18** showed IC_50_ ranging from 0.84 to 3.09 µM against PC3 cell, while the corresponding IC_50_ values for WR1 and LY294002 were 18.88 and 61.35 µM, respectively. Some of the most potent compounds showed nanomolar antiproliferative activity against certain cancer cell lines, such as compound **22** and **25**, which showed IC_50_ values of 100 and 90 nM against HL60, respectively.

**Table 1:** Antiproliferative activity of piperidinylquinoxalines (4–8).

		IC_50_ (µM)^a^				
Cpd.	2-substituenton quinoxaline	PC3	A549	HCT116	HL60	KB
**4**	4-carbamoylpiperidin-1-yl	1.20	26.65	1.57	0.22	13.73
**5**	4-carbamoylpiperidin-1-yl	2.28	27.35	1.20	0.14	11.85
**6**	4-carbamoylpiperidin-1-yl	2.28	16.97	2.55	0.21	10.23
**7**	4-carbamoylpiperidin-1-yl	1.17	12.11	1.61	0.15	4.22
**8**	4-carbamoylpiperidin-1-yl	4.36	10.23	7.13	4.15	6.45
WR1	morpholino	18.88	12.55	5.35	4.47	NT^b^
LY294002	–	61.35	89.65	56.01	9.94	44.76

a The mean value of at least two separate determinations.

b NT: not tested.

**Table 2:** Antiproliferative activity of piperazinylquinoxalines (9–13).

		IC_50_ (µM)^a^				
Cpd.	2-substituenton quinoxaline	PC3	A549	HCT116	HL60	KB
**9**	4-acetylpiperazin-1-yl	2.25	1.02	1.84	2.43	4.42
**10**	4-acetylpiperazin-1-yl	2.27	2.42	2.60	3.28	3.89
**11**	4-acetylpiperazin-1-yl	2.18	18.63	3.66	0.48	10.35
**12**	4-acetylpiperazin-1-yl	3.03	9.79	0.53	0.12	4.30
**13**	4-acetylpiperazin-1-yl	3.17	7.12	4.34	1.78	6.15
WR1	morpholino	18.88	12.55	5.35	4.47	NT^b^
LY294002	–	61.35	89.65	56.01	9.94	44.76

a The mean value of at least two separate determinations.

b NT: not tested.

**Table 3:** Antiproliferative activity of piperazinylquinoxalines (14–53).

		IC_50_ (µM)^a^				
Cpd.	2-substituenton quinoxaline	PC3	A549	HCT116	HL60	KB
**14**	4-methylpiperazin-1-yl	2.94	14.03	2.78	0.77	4.54
**15**	4-methylpiperazin-1-yl	0.84	2.4	1.68	0.69	0.47
**16**	4-methylpiperazin-1-yl	1.55	8.57	2.37	0.54	2.53
**17**	4-methylpiperazin-1-yl	1.01	5.18	2.31	0.28	0.87
**18**	4-methylpiperazin-1-yl	3.09	7.95	8.21	2.75	21.64
**19**	piperazin-1-yl	0.90	12.82	0.99	0.11	8.27
**20**	piperazin-1-yl	1.32	14.39	0.47	0.12	7.26
**21**	piperazin-1-yl	0.68	1.37	0.63	0.16	11.48
**22**	piperazin-1-yl	0.36	0.75	0.19	0.10	9.89
**23**	piperazin-1-yl	2.05	2.79	2.43	1.39	4.58
**24**	3-methylpiperazin-1-yl	1.44	1.36	0.68	0.15	8.73
**25**	3-methylpiperazin-1-yl	2.03	1.60	1.17	0.09	9.18
**26**	3-methylpiperazin-1-yl	0.93	1.65	0.54	0.16	8.53
**27**	3-methylpiperazin-1-yl	0.60	0.57	0.32	0.12	9.46
**28**	3-methylpiperazin-1-yl	2.34	3.45	2.20	1.95	2.03
**29**	4-benzoylpiperazin-1-yl	4.99	5.20	42.36	7.20	1.22
**30**	4-benzoylpiperazin-1-yl	5.81	8.03	6.90	17.28	3.67
**31**	4-benzoylpiperazin-1-yl	3.63	7.43	17.24	6.93	5.65
**32**	4-benzoylpiperazin-1-yl	1.40	3.96	10.08	17.37	3.37
**33**	4-benzoylpiperazin-1-yl	1.77	1.16	8.88	6.95	1.22
**34**	4-(4-chlorobenzoyl)piperazin-1-yl	2.74	3.86	25.38	5.84	0.93
**35**	4-(4-chlorobenzoyl)piperazin-1-yl	3.61	21.57	9.84	6.09	2.84
**36**	4-(4-chlorobenzoyl)piperazin-1-yl	2.43	>50	9.62	5.58	6.03
**37**	4-(4-chlorobenzoyl)piperazin-1-yl	2.89	>50	10.57	6.11	5.09
**38**	4-(4-chlorobenzoyl)piperazin-1-yl	4.05	17.70	8.75	4.44	1.88
**39**	4-(methylsulfonyl)piperazin-1-yl	1.07	2.37	1.21	NT^b^	NT^b^
**40**	4-(methylsulfonyl)piperazin-1-yl	5.35	1.93	1.07	NT^b^	NT^b^
**41**	4-(methylsulfonyl)piperazin-1-yl	2.21	1.32	0.55	NT^b^	NT^b^
**42**	4-(methylsulfonyl)piperazin-1-yl	1.95	1.32	0.41	NT^b^	NT^b^
**43**	4-(methylsulfonyl)piperazin-1-yl	3.30	1.26	0.33	NT^b^	NT^b^
**44**	4-tosylpiperazin-1-yl	2.19	24.26	7.49	6.40	3.08
**45**	4-tosylpiperazin-1-yl	3.48	49.47	1.92	9.28	2.68
**46**	4-tosylpiperazin-1-yl	6.20	>50	14.22	14.95	2.35
**47**	4-tosylpiperazin-1-yl	4.89	19.51	5.07	10.27	2.73
**48**	4-tosylpiperazin-1-yl	3.39	48.23	3.76	9.48	5.17
**49**	4-(3-morpholinopropyl)piperazin-1-yl	5.12	0.73	0.65	NT^b^	NT^b^
**50**	4-(3-morpholinopropyl)piperazin-1-yl	3.83	0.51	0.31	NT^b^	NT^b^
**51**	4-(3-morpholinopropyl)piperazin-1-yl	9.30	2.10	2.59	NT^b^	NT^b^
**52**	4-(3-morpholinopropyl)piperazin-1-yl	1.19	0.34	0.22	NT^b^	NT^b^
**53**	4-(3-morpholinopropyl)piperazin-1-yl	4.80	1.12	0.93	NT^b^	NT^b^
LY^c^	–	61.35	89.65	56.01	9.94	44.76

a The mean value of at least two separate determinations.

b NT: not tested.

c LY: LY294002.

Reversion of the 4-carbamoylpiperidin-1-yl group of compounds **4–8** into a 4-acetylpiperazin-1-yl group resulted in compounds **9–10** with retained inhibitory potency against tested cell lines (Table 2). For instance, compounds **9–10** showed IC_50_ values of 4.42, 3.89, 10.35, 4.30, and 6.15 µM against KB cell, respectively, which were equivalent to that of compounds **4–8** (KB, IC_50_ values of 13.73, 11.85, 10.23, 4.22 and 6.45 µM, respectively).

A view on inhibitory data of compounds **14–28** showed that the existence of a methyl group on 4-position of the piperazinyl ring had little effort on antiproliferative activity. For example, compounds **15** with a 4-methylpiperazin-1-yl group, **20** with a piperazin-1-yl group and **25** with a 3-methylpiperazin-1yl group showed IC_50_ values of 1.68, 0.47 and 1.17 µM, respectively, against HCT116.

Comparison of cytotoxic data in Table 2 and 3 also revealed that compounds **29–33** with a 4-benzoylpiperazin-1-yl group and compounds **34–38** with a 4-(4-chlorobenzoyl)piperazin-1-yl group showed decreased potency than compounds **9–13** with a 4-acetylpiperazin-1-yl group. For example, compound **9** showed an IC_50_ value of 1.84 µM against HCT116, while compounds **29** and **34** showed IC_50_ values of 42.36 and 25.38 µM, respectively, against HCT116. Similarly, compounds **44–48** with a 4-(4-methylphenylsulfonyl)-piperazin-1-yl group showed decreased potency than compounds **39–43** with a 4-(methylsulfonyl)piperazin-1-yl group. For example, compound **43** inhibited A549 with an IC_50_ value of 1.26 µM, while compound **48** inhibited A549 with an IC_50_ value of 48.23 µM. These results indicated that an aryl susbtituent on the 4-piperaziny-1-yl group at the 2-position of the quinoxaline scaffold was unfavorable for antiproliferative activity.

Besides, compounds with a long flexible (4-(3-morpholinopropyl)piperazin-1-yl group (**49–53**) showed potent low micromolar to nanomolar antiproliferative activity against three tested cancer cell lines. For instance, the tested IC_50_ values of compound **52** against PC3, A549 and HCT116 were 1.19, 0.34 and 0.22 µM, respectively.

#### Inhibition of PI3Kα

Selected compounds were then tested for their enzymatic inhibitory activity against PI3Kα using a competitive fluorescence polarization (FP) assay to determine the molecular target of synthesized compounds [21]. As shown in Table 4, compound **4** with a 4-carbamoylpiperidin-1-yl group did not show significant inhibitory activity against PI3Kα (IC_50_ value >10 µM). Most tested piperazinylquinoxaline derivatives showed comparable PI3Kα inhibitory activity with that of WR1 and LY294002. The most potent compounds 2-(piperazin-1-yl)-3-(4-bromophenylsulfonyl)quinoxaline **22** (IC_50_: 40 nM) and 2-(4-(methylsulfonyl)piperazin-1-yl)-3-(4-methoxyphenylsulfonyl)quinoxaline **41** (IC_50_: 24 nM) showed nanomolar inhibitory activity against PI3Kα. Consistent with the result of antiproliferative test, compound **29** with a 4-benzoylpiperazin-1-yl group (IC_50_: >10 µM) and compound **44** with a 4-(4-methylphenylsulfonyl)piperazin-1-yl group (IC_50_: >10 µM) showed less potent PI3Kα inhibitory activity than that of compound **24** with a 3-methylpiperazin-1-yl group (IC_50_: 0.059 µM) and compound **39** with a 4-(methylsulfonyl)piperazin-1-yl group (IC_50_: 1.34 µM). The values of binding efficiency index (BEI), a modified ligand efficiency index based on a molecular weight (MW) scale [22], were calculated for target compounds that exhibited good to potent PI3Kα inhibitory activity to evaluate binding efficiency of these compounds. As shown in Table 5, although most compounds showed BEI values comparable to that of WR1, LY294002 or WR23, no significant improvement in ligand binding efficiency was observed. This analysis based on BEI indicated that further modification with an aim to improve ligand binding efficiency might expedite the optimization process for this series of compounds.

**Table 4:** PI3Kα inhibitory activity of target compounds.

Cpd.	2-substituent on quinoxaline scaffold	4-substituent of phenylsulfonyl moiety	IC_50_ (µM)^a^
**4**	4-carbamoylpiperidin-1-yl	H	>10
**9**	4-acetylpiperazin-1-yl	H	1.087
**14**	4-methylpiperazin-1-yl	H	>10
**15**	4-methylpiperazin-1-yl	methyl	>10
**17**	4-methylpiperazin-1-yl	bromo	4.25
**19**	piperazin-1-yl	H	0.19
**22**	piperazin-1-yl	bromo	0.04
**24**	3-methylpiperazin-1-yl	H	0.059
**25**	3-methylpiperazin-1-yl	methyl	0.65
**26**	3-methylpiperazin-1-yl	methoxy	0.24
**27**	3-methylpiperazin-1-yl	bromo	0.061
**29**	4-benzoylpiperazin-1-yl	H	>10
**34**	4-(4-chlorobenzoyl)piperazin-1-yl	H	2.75
**39**	4-(methylsulfonyl)piperazin-1-yl	H	1.34
**40**	4-(methylsulfonyl)piperazin-1-yl	methyl	0.46
**41**	4-(methylsulfonyl)piperazin-1-yl	methoxy	0.024
**42**	4-(methylsulfonyl)piperazin-1-yl	bromo	0.44
**43**	4-(methylsulfonyl)piperazin-1-yl	fluoro	1.012
**44**	4-(4-methylphenylsulfonyl)piperazin-1-yl	H	>10
**49**	4-(3-morpholinopropyl)piperain-1-yl	H	0.18
**52**	4-(3-morpholinopropyl)piperain-1-yl	bromo	0.20
WR1^c^	morpholino	H	0.44
LY294002^d^	–	–	0.63

a The mean value of at least two separate determinations.

b NT: not tested.

c Reported value, ref. 14.

d Reported value, ref. 16.

**Table 5:** Binding efficiency index (BEI) of selected target compounds.

Cpd.	calculated MW (kDa)	IC_50_ (M)	BEI^a^
**9**	0.396	0.000001087	15.1
**17**	0.448	0.00000425	12.0
**19**	0.354	0.00000019	19.0
**22**	0.434	0.00000004	17.0
**24**	0.368	0.000000059	19.7
**25**	0.382	0.00000065	16.2
**26**	0.398	0.00000024	16.6
**27**	0.448	0.000000061	16.1
**34**	0.492	0.00000275	11.3
**39**	0.432	0.00000134	13.6
**40**	0.466	0.00000046	13.6
**41**	0.462	0.000000024	16.5
**42**	0.512	0.00000044	12.4
**43**	0.450	0.000001012	13.3
**49**	0.481	0.00000018	14.0
**52**	0.561	0.00000020	11.9
WR1	0.355	0.00000044	17.9
LY294002	0.307	0.00000063	20.2
WR23	0.448	0.000000025	17.0

a Binding efficiency index (BEI) = pIC_50_ (M)/MW (kDa).

#### Apoptosis assay

**Figure 5:**
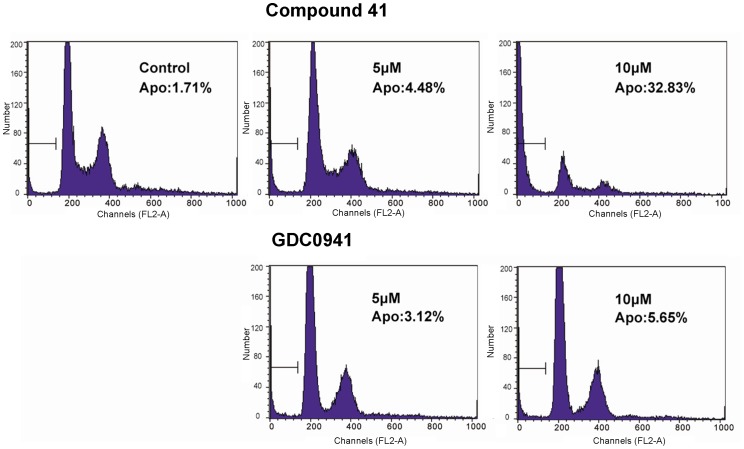
Apoptosis induction of compound 41 and GDC0941 in PC3 cells. After treatment with **41** and GDC0941 (5 or 10 µM) for 24 h, PC3 cells were harvested and detected of apoptosis by flow cytometry using PI apoptosis detection kit. Vertical axes stand for the counts of relative number of PC3 cells; horizontal axes stand for the relative fluorescence light intensity measured as pulse-area (FL2-A).

Piperazinylquinoxaline derivative **41** was further tested for its ability to induce apoptosis in PC3 cells. GDC0941, one of the most advanced PI3K inhibitors revealed so far, was used as the positive control [23] (Fig. 5). With an apoptotic percent of 1.71% of the control, the percent of apoptotic PC3 cells induced by compound **41** and GDC0941 in 5 µM after treatment of 24 h were 4.48% and 3.12%, respectively. The fact that compound **41** showed an apoptotic percent of 32.83% in 10 µM, in comparison with that of 5.85 for GDC0941, indicated the potent apoptosis inductive activity of compound **41**.

#### Cell cycle arrest

**Figure 6:**
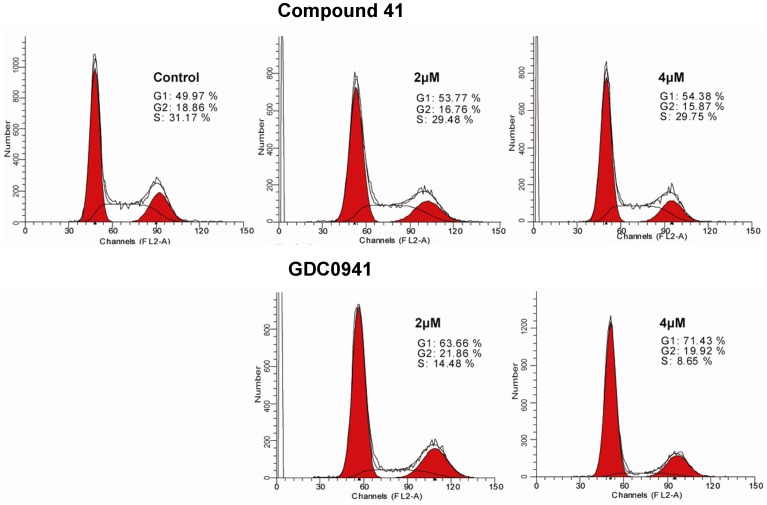
Cell cycle arrest test of compound 41 and GDC0941 in PC3 cells. Vertical axes stand for the counts of relative number of PC3 cells; horizontal axes stand for the relative fluorescence light intensity measured as pulse-area (FL2-A).

Moreover, flow cytometric analysis was performed to determine whether target compounds could induce cell cycle arrest in PC3 cells. GDC0941 was used as the positive control. PC3 cells were treated with compound **41** and GDC0941 in two different concentrations (2 and 4 µM) for 24 h, the results are presented as Figure 6. GDC0941 induced cell cycle arrest in G1 phase with a simultaneous decrease of cells in S phase. Compound **41** showed similar trend while the percent of cell in G1 phase was smaller.

### Molecular Docking Analysis

**Figure 7:**
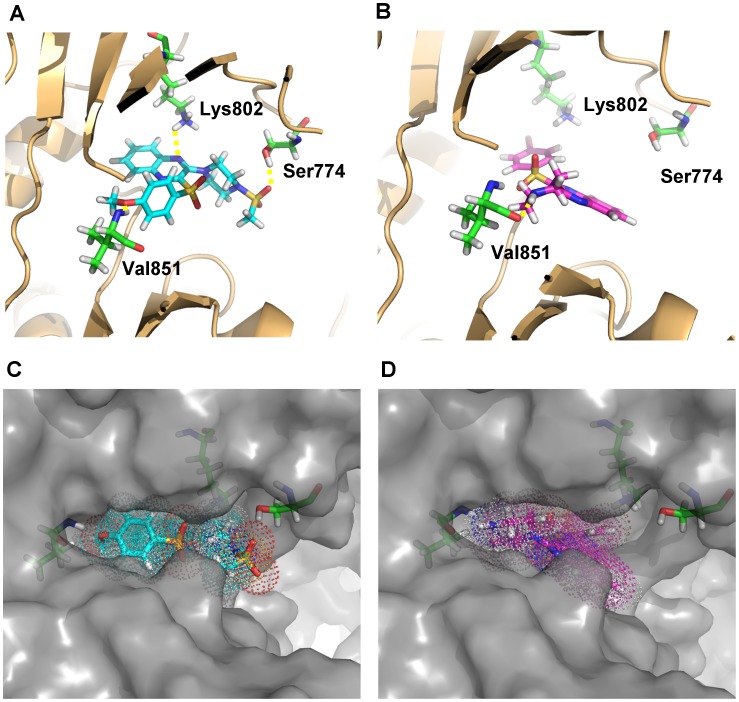
Molecular docking of compounds 41 and 22 with PI3Kα. Compound **41** is shown in cyan backbone, compound **22** is shown in magenta backbone, and selected amino residues are shown in green backbones. (A) Cartoon show of **41** docked into the ATP binding site of PI3Kα; (B) cartoon show of **22**-PI3Kα docking complex; (C) surface show of **41**-PI3Kα docking complex with **41** shown in dotted stick; (D) surface show of **22**- PI3Kα docking complex with **22** shown in dotted stick.

Compounds **41** and **22** that showed the most potent inhibitory activity against PI3Kα were subjected to molecular docking analysis to investigate possible binding mode between target compounds and PI3Ks. Co-crystal structures of mutant PI3Kα with small-molecule inhibitor (PDB ID: 3HHM) was utilized as the template to perform docking analysis [24]. Based on the docking results as shown in Figure 7, compound **41** might form three hydrogen bond interactions with PI3Kα, the methoxy oxygen with the NH of Val851 (distance: 2.1 *Å*), one of the methylsulfonyl oxygen with the OH of Ser774 (distance: 1.9 *Å*), and one of the quinoxaline nitrogen with the NH_2_ of Lys802 (distance 2.4 *Å*) (Fig. 7A); the hydrogen bond interaction with Val882 is probably retained in the PI3Kα-**22** docking complex (Fig. 7B). Although both **41** and **22** have the potential to interact with Val851 through the formation of a hydrogen bond interaction that is believed to be of significant importance for PI3K inhibition [25], **41** and **22** tend to bind with PI3Kα in different modes, the quinoxaline moiety of **41** might bind with an affinity pocket close to Lys802 and its methylsulfonylpiperazinyl moiety extends to the solvent front (Fig. 7C), while the quinoxaline moiety of **22** might extend to the solvent front with its bromophenylsulfonyl moiety binds with the affinity pocket (Fig. 7D).

### Conclusions

Series of novel piperazinylquinixaline derivatives have been identified as PI3Kα inhibitors in this study. Representative compounds 2-(piperazin-1-yl)-3-(4-bromophenylsulfonyl)quinoxaline (**22**) and 2-(4-(methylsulfonyl)piperazin-1-yl)-3-(4-methoxyphenylsulfonyl)quinoxaline (**41**) exhibit low micromolar to nanomolar antiproliferative potency against human cell lines and inhibit PI3Kα with IC_50_ values of 40 and 24 nM, respectively. Compound **41** potently induces apoptosis and appears to have certain effort on cell cycle arrest in G1 phase. Molecular docking analysis shows the possible binding modes between **41** and PI3Ks. Our data hold promise for the development of piperazinylquinoxaline derivatives as PI3K inhibitors for cancer therapy.

## Materials and Methods

### Experimental Methods

#### Reagents and apparatus

Melting points were determined with a B-540 Büchi apparatus and are uncorrected. NMR spectra were recorded on a Brüker 500 (500 MHz) spectrometer at room temperature (chemical shifts are reported in ppm (δ) using TMS as internal standard, coupling constants (J) are in hertz (Hz), and signals are designated as follows: s, singlet; d, doublet; t, triplet; m, multiplet; brs, broad singlet, etc.) Mass spectra (MS), ESI (positive) were recorded on an Esquire-LC-00075 spectrometer. Thin layer chromatography was carried out using plate silica gel F254 (Merck, Damstadt, Germany). All commercially obtained reagents were used as received unless otherwise noticed. All yields are unoptimized and generally represent the result of a single experiment.

#### General procedure for the synthesis of 4–8 (procedure A)

To a microwave vial (2–5 mL) were added 2-chloro-3-(arylsulfonyl)quinoxaline **55–59** (0.1 mmol), *N*-carbamoylpiperidine (0.5 mmol), and isopropyl alcohol (2 mL). The sealed vial was heated at 80°C for 10 min by microwave irradiation in a Biotage™ Initiator Synthesizer using a fixed hold time. The mixture was then cooled to room temperature and the residue obtained after evaporating under vacuum was subjected to purification over silica gel chromatography eluting with PE: EtOAc (4∶1, v/v) to afford target compounds as yellow solid.

#### 2-(4-Carbamoylpiperidin-1-yl)-3-(phenylsulfonyl)Fquinoxaline 4

Yield: 64%; mp: 170–174°C. ^1^H NMR (500 MHz, CDCl_3_): δ 8.00 (d, 2H, *J* = 7.5 Hz, aromatic H), 7.77 (d, 1H, *J* = 8.5 Hz, aromatic H), 7.69-7.62 (m, 3H, aromatic H), 7.55 (t, 2H, *J* = 8.0 Hz, aromatic H), 7.47 (t, 1H, *J* = 7.5 Hz, aromatic H), 5.59 (brs, 1H, NH_2_), 5.40 (brs, 1H, NH_2_), 4.36 (d, 2H, *J* = 13.0 Hz, piperidine H), 3.20 (dt, 2H, *J* = 13.0 and 3.0 Hz, piperidine H), 2.51-2.45 (m, 1H, piperidine H), 2.11-2.04 (m, 4H, piperidine H). ESI-MS (*m/z*): 397 [M+1]^+^.

#### 2-(4-Carbamoylpiperidin-1-yl)-3-(4-methylphenylfsulfonyl)quinoxaline 5

Yield: 53%; mp: 200–203°C. ^1^H NMR (500 MHz, CDCl_3_): δ 7.88 (d, 2H, *J* = 8.0 Hz, aromatic H), 7.76 (d, 1H, *J* = 8.5 Hz, aromatic H), 7.69-7.65 (m, 2H, aromatic H), 7.46 (t, 1H, *J* = 8.5 Hz, aromatic H), 7.32 (d, 2H, *J* = 8.0 Hz, aromatic H), 5.70 (brs, 2H, NH_2_), 4.34 (d, 2H, *J* = 13.0 Hz, piperidine H), 3.17 (dt, 2H, *J* = 13.0 and 3.0 Hz, piperidine H), 2.48-2.42 (m, 4H, piperidine H and CH_3_), 2.08-2.02 (m, 4H, piperidine H). ESI-MS (*m/z*): 411 [M+1]^+^.

#### 2-(4-Carbamoylpiperidin-1-yl)-3-(4-methoxyphenylsulfonyl)quinoxaline 6

Yield: 42%; mp: 185–187°C. ^1^H NMR (500 MHz, CDCl_3_): δ 7.94 (d, 2H, *J* = 8.5 Hz, aromatic H), 7.76 (d, 1H, *J* = 8.0 Hz, aromatic H), 7.70-7.65 (m, 2H, aromatic H), 7.47 (t, 1H, *J* = 7.5 Hz, aromatic H), 7.00 (d, 2H, *J* = 8.5 Hz, aromatic H), 5.60 (brs, 1H, NH_2_), 5.39 (brs, 1H, NH_2_), 4.36 (d, 2H, *J* = 13.0 Hz, piperidine H), 3.88 (s, 3H, OCH_3_), 3.18 (dt, 2H, *J* = 13.0 and 3.0 Hz, piperidine H), 2.49-2.46 (m, 1H, piperidine H), 2.10-2.05 (m, 4H, piperidine H). ESI-MS (*m/z*): 427 [M+1]^+^.

#### 2-(4-Carbamoylpiperidin-1-yl)-3-(4-bromophenylsulfonyl)quinoxaline 7

Yield: 61%; mp: 209–210°C. ^1^H NMR (500 MHz, CDCl_3_): δ 7.94 (d, 1H, *J* = 9.0 Hz, aromatic H), 7.86 (d, 1H, *J* = 8.0 Hz, aromatic H), 7.77-7.75 (m, 1H, aromatic H), 7.70-7.64 (m, 3H, aromatic H), 7.48-7.45 (m, 1H, aromatic H), 7.00 (d, 1H, *J* = 9.0 Hz, aromatic H), 5.60 (brs, 1H, NH_2_), 5.43 (brs, 1H, NH_2_), 4.37 (d, 2H, *J* = 13.0 Hz, piperidine H), 3.21-3.16 (m, 2H, piperidine H), 2.49-2.45 (m, 1H, piperidine H), 2.11-2.05 (m, 4H, piperidine H). ESI-MS (*m/z*): 477 [M+1]^+^.

#### 2-(4-Carbamoylpiperidin-1-yl)-3-(4-fluorophenylsulfonyl)quinoxaline 8

Yield: 68%; mp: 198–202°C. ^1^H NMR (500 MHz, CDCl_3_): δ 8.03 (d, 2H, *J* = 8.5 Hz, aromatic H), 7.77 (d, 1H, *J* = 8.5 Hz, aromatic H), 7.69 (t, 1H, *J* = 8.5 Hz, aromatic H), 7.64 (d, 1H, *J* = 7.5 Hz, aromatic H), 7.47 (dt, 1H, *J* = 8.0 and 1.0 Hz, aromatic H), 7.23 (t, 2H, *J* = 8.0 Hz, aromatic H), 5.60 (brs, 1H, NH_2_), 5.47 (brs, 1H, NH_2_), 4.38 (d, 2H, *J* = 13.0 Hz, piperidine H), 3.21 (dt, 2H, *J* = 13.0 and 3.0 Hz, piperidine H), 2.50-2.45 (m, 1H, piperidine H), 2.11-2.04 (m, 4H, piperidine H). ESI-MS (*m/z*): 415 [M+1]^+^.

#### General procedure for the synthesis of 9–13

Similar with procedure A, but *N*-carbamoylpiperazine was replaced by *N*-acetylpiperazine. Target compounds were obtained as yellow solid.

#### 2-(4-Acetylpiperazin-1-yl)-3-(phenylsulfonyl)quinoxaline 9

Yield: 38%; mp: 144–146°C. ^1^H NMR (500 MHz, CDCl_3_): δ 8.01-8.00 (m, 2H, aromatic H), 7.81-7.79 (m, 1H, aromatic H), 7.71-7.67 (m, 3H, aromatic H), 7.55 (m, 2H, aromatic H), 7.50 (m, 1H, aromatic H), 3.89 (m, 2H, piperidine H), 3.76-3.71 (m, 4H, piperidine H), 3.67 (m, 2H, piperidine H), 2.18 (s, 3H, CH_3_). ESI-MS (*m/z*): 397 [M+1]^+^.

#### 2-(4-Acetylpiperazin-1-yl)-3-(4-methylphenylsulfonyl)quinoxaline 10

Yield: 36%; mp: 148–150°C. ^1^H NMR (500 MHz, CDCl_3_): δ 7.89 (d, 2H, *J* = 8.0 Hz, aromatic H), 7.80 (d, 1H, *J* = 8.0 Hz, aromatic H), 7.72 (t, 2H, *J* = 7.5 Hz, aromatic H), 7.52 (t, 1H, *J* = 7.5 Hz, aromatic H), 7.34 (d, 2H, *J* = 8.5 Hz, aromatic H), 3.90 (t, 2H, *J* = 5.0 Hz, piperidine H), 3.78-3.73 (m, 4H, piperidine H), 3.67 (t, 2H, *J* = 5.0 Hz, piperidine H), 2.46 (s, 3H, aromatic CH_3_), 2.18 (s, 3H, CH_3_). ESI-MS (*m/z*): 411 [M+1]^+^.

#### 2-(4-Acetylpiperazin-1-yl)-3-(4-methoxyphenylsulfonyl)quinoxaline 11

Yield: 39%; mp: 160–162°C. ^1^H NMR (500 MHz, CDCl_3_): δ 7.95 (d, 2H, *J* = 9.0 Hz, aromatic H), 7.80 (d, 1H, *J* = 8.5 Hz, aromatic H), 7.72-7.70 (m, 2H, aromatic H), 7.52 (dt, 1H, *J* = 8.0 and 1.0 Hz, aromatic H), 7.01 (d, 2H, *J* = 9.0 Hz, aromatic H), 3.91-3.89 (m, 5H, piperidine H and aromatic OCH_3_), 3.78-3.73 (m, 4H, piperidine H), 3.68 (t, 2H, *J* = 5.0 Hz, piperidine H), 2.18 (s, 3H, CH_3_). ESI-MS (*m/z*): 427 [M+1]^+^.

#### 2-(4-Acetylpiperazin-1-yl)-3-(4-bromophenylsulfonyl)quinoxaline 12

Yield: 39%; mp: 174–178°C. ^1^H NMR (500 MHz, CDCl_3_): δ 7.90-7.86 (m, 2H, aromatic H), 7.81-7.79 (m, 1H, aromatic H), 7.72-7.66 (m, 4H, aromatic H), 7.54-7.50 (m, 1H, aromatic H), 3.90 (m, 2H, piperidine H), 3.77 (m, 4H, piperidine H), 3.72-3.70 (m, 2H, piperidine H), 2.18 (s, 3H, CH_3_). ESI-MS (*m/z*): 477 [M+1]^+^.

#### 2-(4-Acetylpiperazin-1-yl)-3-(4-fluorophenylsulfonyl)quinoxaline 13

Yield: 56%; mp: 108–112°C. ^1^H NMR (500 MHz, CDCl_3_): δ 8.04 (d, 2H, *J* = 8.5 Hz, aromatic H), 7.81 (dd, 1H, *J* = 8.5 and 1.0 Hz, aromatic H), 7.73 (dt, 1H, *J* = 8.5 and 1.5 Hz, aromatic H), 7.66 (dd, 1H, *J* = 8.0 and 1.0 Hz, aromatic H), 7.52 (dt, 1H, *J* = 8.5 and 1.5 Hz, aromatic H), 7.25 (dt, 2H, *J* = 8.5 and 1.5 Hz, aromatic H), 3.92 (t, 2H, *J* = 5.0 Hz, piperidine H), 3.77 (m, 4H, piperidine H), 3.71 (t, 2H, *J* = 5.0 Hz, piperidine H), 2.18 (s, 3H, CH_3_). ESI-MS (*m/z*): 415 [M+1]^+^.

#### General procedure for the synthesis of 14–18

Similar with procedure A, but *N*-carbamoylpiperazine was replaced by *N*-methylpiperazine. Target compounds were obtained as yellow solid.

#### 2-(4-Methylpiperazin-1-yl)-3-(phenylsulfonyl)quinoxaline 14

Yield: 74%; mp: 154–157°C. ^1^H NMR (500 MHz, CDCl_3_): δ 8.01 (d, 2H, *J* = 8.0 Hz, aromatic H), 7.77 (d, 1H, *J* = 8.0 Hz, aromatic H), 7.67-7.61 (m, 3H, aromatic H), 7.54 (t, 2H, *J* = 7.5 Hz, aromatic H), 7.44 (t, 1H, *J* = 8.0 Hz, aromatic H), 3.90 (t, 4H, *J* = 4.5 Hz, piperidine H), 2.71 (t, 4H, *J* = 4.5 Hz, piperidine H), 2.40 (s, 3H, piperidine CH_3_). ESI-MS (*m/z*): 369 [M+1]^+^.

#### 2-(4-Methylpiperazin-1-yl)-3-(4-methylphenylsulfonyl)quinoxaline 15

Yield: 74%; mp: 127–130°C. ^1^H NMR (500 MHz, CDCl_3_): δ 7.89 (d, 2H, *J* = 6.0 Hz, aromatic H), 7.76 (d, 1H, *J* = 6.0 Hz, aromatic H), 7.67-7.64 (m, 2H, aromatic H), 7.43 (t, 1H, *J* = 6.0 Hz, aromatic H), 7.32-7.31 (m, 2H, aromatic H), 3.80 (t, 4H, *J* = 4.5 Hz, piperidine H), 2.70 (t, 4H, *J* = 4.5 Hz, piperidine H), 2.45 (s, 3H, aromatic CH_3_), 2.40 (s, 3H, piperidine CH_3_). ESI-MS (*m/z*): 383 [M+1]^+^.

#### 2-(4-Methylpiperazin-1-yl)-3-(4-methoxyphenylsulfonyl)quinoxaline 16

Yield: 84%; mp: 121–124°C. ^1^H NMR (500 MHz, CDCl_3_): δ 7.95 (d, 2H, *J* = 8.5 Hz, aromatic H), 7.77 (d, 1H, *J* = 9.0 Hz, aromatic H), 7.67-7.64 (m, 2H, aromatic H), 7.45 (t, 1H, *J* = 8.5 Hz, aromatic H), 7.00 (d, 2H, *J* = 9.0 Hz, aromatic H), 3.89 (s, 3H, aromatic OCH_3_), 3.81 (t, 4H, *J* = 4.5 Hz, piperidine H), 2.75 (t, 4H, *J* = 4.5 Hz, piperidine H), 2.43 (s, 3H, piperidine CH_3_). ESI-MS (*m/z*): 399 [M+1]^+^.

#### 2-(4-Methylpiperazin-1-yl)-3-(4-bromophenylsulfonyl)quinoxaline 17

Yield: 69%; mp: 141–142°C. ^1^H NMR (500 MHz, CDCl_3_): δ 7.87 (d, 2H, *J* = 8.5 Hz, aromatic H), 7.77 (d, 1H, *J* = 8.0 Hz, aromatic H), 7.68-7.66 (m, 3H, aromatic H), 7.63 (d, 1H, *J* = 8.5 Hz, aromatic H), 7.46 (d, 1H, *J* = 7.5 Hz, aromatic H), 3.81 (t, 4H, *J* = 4.5 Hz, piperidine H), 2.71 (t, 4H, *J* = 4.5 Hz, piperidine H), 2.40 (s, 3H, piperidine CH_3_). ESI-MS (*m/z*): 449 [M+1]^+^.

#### 2-(4-Methylpiperazin-1-yl)-3-(4-fluorophenylsulfonyl)quinoxaline 18

Yield: 85%; mp: 147–148°C. ^1^H NMR (500 MHz, CDCl_3_): δ 8.03 (dd, 2H, *J* = 8.5 and 2.0 Hz, aromatic H), 7.77 (d, 1H, *J* = 8.5 Hz, aromatic H), 7.69 (dt, 1H, *J* = 7.0 and 1.5 Hz, aromatic H), 7.62 (d, 1H, *J* = 8.5 Hz, aromatic H), 7.46 (dt, 1H, *J* = 7.0 and 1.5 Hz, aromatic H), 7.23 (t, 2H, *J* = 8.5 Hz, aromatic H), 3.82 (m, 4H, piperidine H), 2.74 (m, 4H, piperidine H), 2.43 (s, 3H, piperidine CH_3_). ESI-MS (*m/z*): 387 [M+1]^+^.

#### General procedure for the synthesis of 19–23

Similar with procedure A, but *N*-carbamoylpiperazine was replaced by piperazine.

#### 2-(Piperazin-1-yl)-3-(phenylsulfonyl)quinoxaline 19

Yellow mucous substance. Yield: 64%; ^1^H NMR (500 MHz, CDCl_3_): δ 8.00 (d, 2H, *J* = 8.0 Hz, aromatic H), 7.79 (d, 1H, *J* = 9.0 Hz, aromatic H), 7.69-7.62 (m, 3H, aromatic H), 7.55 (t, 2H, *J* = 8.0 Hz, aromatic H), 7.47 (dt, 1H, *J* = 8.5 and 1.0 Hz, aromatic H), 3.73 (t, 4H, *J* = 4.5 Hz, piperidine H), 3.17 (t, 4H, *J* = 6.5 Hz, piperidine H). ESI-MS (*m/z*): 355 [M+1]^+^.

#### 2-(Piperazin-1-yl)-3-(4-methylphenylsulfonyl)quinoxaline 20

Orange-yellow mucous substance. Yield: 73%; ^1^H NMR (500 MHz, CDCl_3_): δ 7.89 (d, 2H, *J = *8.0 Hz, aromatic H), 7.77 (d, 1H, *J = *8.0 Hz, aromatic H), 7.68 (t, 2H, *J = *8.0 Hz, aromatic H), 7.46 (t, 1H, *J = *7.5 Hz, aromatic H), 7.32 (d, 1H, *J = *8.5 Hz, aromatic H), 3.71 (t, 4H, *J = *4.5 Hz, piperidine H), 3.16 (t, 4H, *J = *4.5 Hz, piperidine H), 2.44 (s, 3H, CH_3_). ESI-MS (*m/z*): 369 [M+1]^+^.

#### 2-(Piperazin-1-yl)-3-(4-methoxyphenylsulfonyl)quinoxaline 21

Yellow solid. Yield: 45%; mp: 142–144°C. ^1^H NMR (500 MHz, CDCl_3_): δ 7.95 (d, 2H, *J = *9.0 Hz, aromatic H), 7.77 (d, 1H, *J = *8.0 Hz, aromatic H), 7.69-7.65 (m, 2H, aromatic H), 7.47 (dt, 1H, *J = *8.0 and 1.0 Hz, aromatic H), 7.00 (d, 2H, *J = *8.5 Hz, aromatic H), 3.88 (s, 3H, OCH_3_), 3.73 (t, 4H, *J = *4.5 Hz, piperidine H), 3.17 (t, 4H, *J = *4.5 Hz, piperidine H). ESI-MS (*m/z*): 385 [M+1]^+^.

#### 2-(Piperazin-1-yl)-3-(4-bromophenylsulfonyl)quinoxaline 22

Yellow solid. Yield: 90%; mp: 92–96°C. ^1^H NMR (500 MHz, CDCl_3_): δ 7.94 (d, 2H, *J = *8.5 Hz, aromatic H), 7.79 (d, 1H, *J = *8.5 Hz, aromatic H), 7.72-7.65 (m, 4H, aromatic H), 7.50 (t, 1H, *J = *8.0 Hz, aromatic H), 3.81 (t, 4H, *J = *4.5 Hz, piperidine H), 3.25 (t, 4H, *J = *4.5 Hz, piperidine H). ESI-MS (*m/z*): 435 [M+1]^+^.

#### 2-(Piperazin-1-yl)-3-(4-fluorophenylsulfonyl)quinoxaline 23

Orange-yellow mucous substance. Yield: 81%; ^1^H NMR (500 MHz, CDCl_3_): δ 8.04 (d, 2H, *J = *9.0 Hz, aromatic H), 7.79 (d, 1H, *J = *8.5 Hz, aromatic H), 7.71 (dt, 1H, *J = *8.0 and 1.0 Hz, aromatic H), 7.65 (d, 1H, *J = *8.0 Hz, aromatic H), 7.49 (dt, 2H, *J = *8.0 and 1.0 Hz, aromatic H), 7.24 (t, 2H, *J = *8.5 Hz, aromatic H), 3.82 (t, 4H, *J = *4.5 Hz, piperidine H), 3.28 (t, 4H, *J = *4.5 Hz, piperidine H). ESI-MS (*m/z*): 373 [M+1]^+^.

#### General procedure for the synthesis of 24–28

Similar with procedure A, but *N*-carbamoylpiperazine was replaced by 2-methylpiperazine.

#### 2-(3-Methylpiperazin-1-yl)-3-(phenylsulfonyl)quinoxaline 24

Orange-yellow mucous substance. Yield: 91%; ^1^H NMR (500 MHz, CDCl_3_): δ 7.99 (d, 2H, *J = *8.5 Hz, aromatic H), 7.76 (d, 1H, *J = *8.0 Hz, aromatic H), 7.67-7.61 (m, 3H, aromatic H), 7.53 (t, 2H, *J = *8.0 Hz, aromatic H), 7.44 (dt, 1H, *J = *8.0 and 1.5 Hz, aromatic H), 4.24 (dt, 2H, *J = *11.0 and 2.0 Hz, piperidine H), 3.22-3.16 (m, 4H, piperidine H), 2.84-2.80 (m, 1H, piperidine H), 1.19 (d, 3H, *J = *6.0 Hz, piperidine CH_3_). ESI-MS (*m/z*): 369 [M+1]^+^.

#### 2-(3-Methylpiperazin-1-yl)-3-(4-methylphenylsulfonyl)quinoxaline 25

Yellow solid. Yield: 74%; mp: 136–138°C. ^1^H NMR (500 MHz, CDCl_3_): δ 7.88 (d, 2H, *J = *8.0 Hz, aromatic H), 7.78 (d, 1H, *J = *8.0 Hz, aromatic H), 7.69-7.66 (m, 2H, aromatic H), 7.47 (t, 1H, *J = *8.0 Hz, aromatic H), 7.33 (d, 2H, *J = *8.0 Hz, aromatic H), 4.24-4.22 (m, 2H, piperidine H), 3.31-3.27 (m, 4H, piperidine H), 2.96 (t, 1H, *J = *11.0 Hz, piperidine H), 2.44 (s, 3H, aromatic CH_3_), 1.29 (d, 3H, *J = *6.0 Hz, piperidine CH_3_). ESI-MS (*m/z*): 383 [M+1]^+^.

#### 2-(3-Methylpiperazin-1-yl)-3-(4-methoxyphenylsulfonyl)quinoxaline 26

Yellow solid. Yield: 86%; mp: 130–133°C. ^1^H NMR (500 MHz, CDCl_3_): δ 7.94 (d, 2H, *J = *9.0 Hz, aromatic H), 7.76 (d, 1H, *J = *8.5 Hz, aromatic H), 7.68-7.64 (m, 2H, aromatic H), 7.46 (dt, 1H, *J = *8.5 and 1.5 Hz, aromatic H), 6.99 (d, 2H, *J = *8.5 Hz, aromatic H), 4.25-4.21 (m, 2H, piperidine H), 3.87 (s, 3H, aromatic OCH_3_), 3.29-3.17 (m, 4H, piperidine H), 2.89-2.85 (m, 1H, piperidine H), 1.24 (d, 3H, *J = *6.0 Hz, piperidine CH_3_). ESI-MS (*m/z*): 399 [M+1]^+^.

#### 2-(3-Methylpiperazin-1-yl)-3-(4-bromophenylsulfonyl)quinoxaline 27

Yellow solid. Yield: 88%; mp: 158–160°C. ^1^H NMR (500 MHz, CDCl_3_): δ 7.85 (d, 2H, *J = *8.5 Hz, aromatic H), 7.76 (d, 1H, *J = *8.5 Hz, aromatic H), 7.67-7.64 (m, 3H, aromatic H), 7.62 (dd, 1H, *J = *8.5 and 0.5 Hz, aromatic H), 7.45 (dt, 1H, *J = *8.0 and 1.0 Hz, aromatic H), 4.24-4.20 (m, 2H, piperidine H), 3.24-3.17 (m, 4H, piperidine H), 2.86-2.82 (m, 1H, piperidine H), 1.20 (d, 3H, *J = *6.0 Hz, piperidine CH_3_). ESI-MS (*m/z*): 449 [M+1]^+^.

#### 2-(3-Methylpiperazin-1-yl)-3-(4-fluorophenylsulfonyl)quinoxaline 28

Yellow solid. Yield: 70%; mp: 119–123°C. ^1^H NMR (500 MHz, CDCl_3_): δ 8.02 (d, 2H, *J = *9.0 Hz, aromatic H), 7.82 (d, 1H, *J = *8.5 Hz, aromatic H), 7.73 (t, 1H, *J = *8.5 Hz, aromatic H), 7.67 (d, 1H, *J = *8.5 Hz, aromatic H), 7.53 (t, 1H, *J = *7.5 Hz, aromatic H), 7.25 (t, 2H, *J = *8.5 Hz, aromatic H), 4.29 (d, 2H, *J = *12.0 Hz, piperidine H), 3.62-3.59 (m, 1H, piperidine H), 3.56 (t, 2H, *J = *12.0 Hz, piperidine H), 3.41 (t, 1H, *J = *11.0 Hz, piperidine H), 3.32-3.28 (m, 1H, piperidine H), 1.47 (d, 3H, *J = *6.0 Hz, piperidine CH_3_). ESI-MS (*m/z*): 387 [M+1]^+^.

#### General procedure for the synthesis of 29–33

Similar with procedure A, but *N*-carbamoylpiperazine was replaced by *N*-benzoylpiperazine. Target compounds were obtained as yellow solid.

#### 2-(4-Benzoylpiperazin-1-yl)-3-(phenylsulfonyl)quinoxaline 29

Yield: 31%; mp: 224–226°C. ^1^H NMR (500 MHz, CDCl_3_): δ 8.01 (d, 2H, *J = *7.5 Hz, aromatic H), 7.81 (dd, 1H, *J = *8.5 and 1.0 Hz, aromatic H), 7.72-7.65 (m, 3H, aromatic H), 7.57 (t, 2H, *J = *8.0 Hz, aromatic H), 7.52 (dt, 1H, *J = *8.5 and 1.5 Hz, aromatic H), 7.48-7.44 (m, 5H, aromatic H), 4.11-4.04 (m, 2H, piperazine H), 3.84-3.65 (m, 6H, piperazine H). ESI-MS (*m/z*): 459 [M+1]^+^.

#### 2-(4-Benzoylpiperazin-1-yl)-3-(4-methylphenylsulfonyl)quinoxaline 30

Yield: 35%; mp: 182–185°C. ^1^H NMR (500 MHz, CDCl_3_): δ7.89 (d, 2H, *J = *8.5 Hz, aromatic H), 7.81 (d, 1H, *J = *8.5 Hz, aromatic H), 7.72-7.69 (m, 2H, aromatic H), 7.52 (dt, 1H, *J = *8.0 and 1.5 Hz, aromatic H), 7.48-7.44 (m, 5H, aromatic H), 7.34-7.33 (d, 2H, *J = *7.5 Hz, aromatic H), 4.11-4.04 (m, 2H, piperazine H), 3.81-3.64 (m, 6H, piperazine H), 2.46 (s, 3H, aromatic CH_3_). ESI-MS (*m/z*): 473 [M+1]^+^.

#### 2-(4-Benzoylpiperazin-1-yl)-3-(4-methoxyphenylsulfonyl)quinoxaline 31

Yield: 63%; mp: 166–169°C. ^1^H NMR (500 MHz, CDCl_3_): δ 7.94 (d, 2H, *J = *9.0 Hz, aromatic H), 7.80 (d, 1H, *J = *8.0 Hz, aromatic H), 7.72-7.68 (t, 2H, *J = *8.0 Hz, aromatic H), 7.52-7.43 (m, 6H, aromatic H), 7.01-6.99 (d, 2H, *J = *8.5 Hz, aromatic H), 4.11-4.03 (m, 2H, piperazine H), 3.90 (s, 3H, aromatic OCH_3_), 3.85-3.63 (m, 6H, piperazine H). ESI-MS (*m/z*): 489 [M+1]^+^.

#### 2-(4-Benzoylpiperazin-1-yl)-3-(4-bromophenylsulfonyl)quinoxaline 32

Yield: 50%; mp: 204–205°C. ^1^H NMR (500 MHz, CDCl_3_): δ 7.87 (d, 2H, *J = *8.5 Hz, aromatic H), 7.81 (d, 1H, *J = *8.5 Hz, aromatic H), 7.74-7.66 (m, 4H, aromatic H), 7.52 (dt, 1H, *J = *7.0 and 1.0 Hz, aromatic H), 7.47-7.45 (m, 5H, aromatic H), 4.12-4.05 (m, 2H, piperazine H), 3.84-3.65 (m, 6H, piperazine H). ESI-MS (*m/z*): 539 [M+1]^+^.

#### 2-(4-Benzoylpiperazin-1-yl)-3-(4-fluorophenylsulfonyl)quinoxaline 33

Yield: 74%; mp: 200–202°C. ^1^H NMR (500 MHz, CDCl_3_): δ 8.04 (dd, 2H, *J = *9.0 and 5.0 Hz, aromatic H), 7.81 (d, 1H, *J = *8.5 Hz, aromatic H), 7.74 (dt, 1H, *J = *8.5 and 1.0 Hz, aromatic H), 7.67 (d, 1H, *J = *7.5 Hz, aromatic H), 7.53 (dt, 1H, *J = *8.5 and 1.0 Hz, aromatic H), 7.48-7.43 (m, 5H, aromatic H), 7.25-7.22 (t, 2H, *J = *8.5 Hz, aromatic H) 4.11-4.10 (m, 2H, piperazine H), 3.82-3.70 (m, 6H, piperazine H), ESI-MS (*m/z*): 477 [M+1]^+^.

#### General procedure for the synthesis of 34–38

Similar with procedure A, but *N*-carbamoylpiperazine was replaced by *N*-(4-chlorobenzoyl)piperazine. Target compounds were obtained as yellow solid.

#### 2-(4-(4-Chlorobenzoyl)piperazin-1-yl)-3-(phenylsulfonyl)quinoxaline 34

Yield: 32%; mp: 217–219°C. ^1^H NMR (500 MHz, CDCl_3_): δ 8.01 (d, 2H, *J = *7.0 Hz, aromatic H), 7.81 (d, 1H, *J = *8.0 Hz, aromatic H), 7.73 (t, 1H, *J = *7.5 Hz, aromatic H), 7.69-7.65 (m, 2H, aromatic H), 7.57-7.54 (t, 2H, *J = *7.5 Hz, aromatic H), 7.51 (t, 1H, *J = *7.5 Hz, aromatic H), 7.42 (s, 4H, aromatic H), 4.11-4.07 (m, 2H, piperazine H), 3.78-3.65 (m, 6H, piperazine H). ESI-MS (*m/z*): 493 [M+1]^+^.

#### 2-(4-(4-Chlorobenzoyl)piperazin-1-yl)-3-(4-methylphenylsulfonyl)quinoxaline 35

Yield: 32%; mp: 188–190°C. ^1^H NMR (500 MHz, CDCl_3_): δ 7.89 (d, 2H, *J = *8.0 Hz, aromatic H), 7.81 (d, 1H, *J = *8.5 Hz, aromatic H), 7.72-7.70 (m, 2H, aromatic H), 7.53 (t, 1H, *J = *8.0 Hz, aromatic H), 7.42 (s, 4H, aromatic H), 7.35 (d, 2H, *J = *8.0 Hz, aromatic H), 4.11-4.04 (m, 2H, piperazine H), 3.80-3.64 (m, 6H, piperazine H), 2.46 (s, 3H, aromatic CH_3_). ESI-MS (*m/z*): 507 [M+1]^+^.

#### 2-(4-(4-Chlorobenzoyl)piperazin-1-yl)-3-(4-methoxyphenylsulfonyl)quinoxaline 36

Yield: 44%; mp: 179°C. ^1^H NMR (500 MHz, CDCl_3_): δ 7.95 (d, 2H, *J = *9.5 Hz, aromatic H), 7.80 (d, 1H, *J = *9.0 Hz, aromatic H), 7.73 (t, 2H, *J = *7.5 Hz, aromatic H), 7.53 (t, 1H, *J = *7.5 Hz, aromatic H), 7.43 (s, 4H, aromatic H), 7.02 (d, 2H, *J = *9.0 Hz, aromatic H), 4.11-4.07 (m, 2H, piperazine H), 3.90 (s, 3H, OCH_3_), 3.78-3.69 (m, 6H, piperazine H). ESI-MS (*m/z*): 523 [M+1]^+^.

#### 2-(4-(4-Chlorobenzoyl)piperazin-1-yl)-3-(4-bromophenylsulfonyl)quinoxaline 37

Yield: 26%; mp: 214–216°C. ^1^H NMR (500 MHz, CDCl_3_): δ 7.87 (d, 2H, *J = *8.5 Hz, aromatic H), 7.81 (d, 1H, *J = *8.0 Hz, aromatic H), 7.74-7.67 (m, 4H, aromatic H), 7.54 (dt, 1H, *J = *7.5 and 1.0 Hz, aromatic H), 7.43 (s, 4H, aromatic H), 4.10-4.05 (m, 2H, piperazine H), 3.80-3.66 (m, 6H, piperazine H). ESI-MS (*m/z*): 573 [M+1]^+^.

#### 2-(4-(4-Chlorobenzoyl)piperazin-1-yl)-3-(4-fluorophenylsulfonyl)quinoxaline 38

Yield: 38%; mp: 214–215°C. ^1^H NMR (500 MHz, CDCl_3_): δ8.04 (dd, 2H, *J = *9.0 and 5.0 Hz, aromatic H), 7.81 (d, 1H, *J = *7.5 Hz, aromatic H), 7.74 (dt, 1H, *J = *8.5 and 1.0 Hz, aromatic H), 7.67 (d, 1H, *J = *8.5 Hz, aromatic H), 7.53 (dt, 1H, *J = *8.5 and 1.0 Hz, aromatic H), 7.43 (s, 4H, aromatic H), 7.26-7.22 (t, 2H, *J = *8.5 Hz, aromatic H) 4.12-4.08 (m, 2H, piperazine H), 3.81-3.70 (m, 6H, piperazine H). ESI-MS (*m/z*): 511 [M+1]^+^.

#### General procedure for the synthesis of 39–43

Similar with procedure A, but *N*-carbamoylpiperazine was replaced by *N*-(methylsulfonyl)piperazine. Target compounds were obtained as yellow solid.

#### 2-(4-(Methylsulfonyl)piperazin-1-yl)-3-(phenylsulfonyl)quinoxaline 39

Yield: 47%; mp: 182–185°C. ^1^H NMR (500 MHz, CDCl_3_): δ 8.01 (d, 2H, *J = *7.5 Hz, aromatic H), 7.83 (d, 1H, *J = *8.5 Hz, aromatic H), 7.72-7.64 (m, 3H, aromatic H), 7.58 (t, 2H, *J = *8.0 Hz, aromatic H), 7.52 (dt, 1H, *J = *8.0 and 1.0 Hz, aromatic H), 3.84 (t, 4H, *J = *4.5 Hz, piperazine H), 3.55 (t, 4H, *J = *4.5 Hz, piperazine H), 2.87 (s, 3H, CH_3_). ESI-MS (*m/z*): 433 [M+1]^+^.

#### 2-(4-(Methylsulfonyl)piperazin-1-yl)-3-(4-methylphenylsulfonyl)quinoxaline 40

Yield: 43%; mp: 178–180°C. ^1^H NMR (500 MHz, CDCl_3_): δ 7.89 (d, 2H, *J = *8.5 Hz, aromatic H), 7.82 (d, 1H, *J = *8.0 Hz, aromatic H), 7.73 (t, 1H, *J = *8.0 Hz, aromatic H), 7.69 (d, 1H, *J = *8.5 Hz, aromatic H), 7.52 (t, 1H, *J = *8.0 Hz, aromatic H), 7.36 (d, 2H, *J = *8.0 Hz, aromatic H), 3.84 (t, 4H, *J = *4.5 Hz, piperazine H), 3.55 (t, 4H, *J = *4.5 Hz, piperazine H), 2.87 (s, 3H, CH_3_), 2.47 (s, 3H, aromatic CH_3_). ESI-MS (*m/z*): 467 [M+1]^+^.

#### 2-(4-(Methylsulfonyl)piperazin-1-yl)-3-(4-methoxyphenylfsulfonyl)quinoxaline 41

Yield: 38%; mp: 177–179°C. ^1^H NMR (500 MHz, CDCl_3_): δ 7.94 (d, 2H, *J = *8.5 Hz, aromatic H), 7.82 (d, 1H, *J = *8.0 Hz, aromatic H), 7.72-7.68 (m, 2H, aromatic H), 7.52 (t, 1H, *J = *8.0 Hz, aromatic H), 7.03 (d, 2H, *J = *8.5 Hz, aromatic H), 3.90 (s, 3H, aromatic OCH_3_), 3.84 (t, 4H, *J = *4.5 Hz, piperazine H), 3.55 (t, 4H, *J = *4.5 Hz, piperazine H), 2.88 (s, 3H, CH_3_), ESI-MS (*m/z*): 463 [M+1]^+^.

#### 2-(4-(Methylsulfonyl)piperazin-1-yl)-3-(4-bromophenylsulfonyl)quinoxaline 42

Yield: 42%; mp: 194–196°C. ^1^H NMR (500 MHz, CDCl_3_): δ 7.87 (d, 2H, *J = *8.5 Hz, aromatic H), 7.83 (d, 1H, *J = *7.5 Hz, aromatic H), 7.75 (dd, 1H, *J = *7.0 and 1.0 Hz, aromatic H), 7.72 (d, 1H, *J = *8.5 Hz, aromatic H), 7.67 (d, 2H, *J = *8.5 Hz, aromatic H), 7.54 (dt, 1H, *J = *8.0 and 1.5 Hz, aromatic H), 3.85 (t, 4H, *J = *4.5 Hz, piperazine H), 3.55 (t, 4H, *J = *4.5 Hz, piperazine H), 2.87 (s, 3H, CH_3_). ESI-MS (*m/z*): 513 [M+1]^+^.

#### 2-(4-(Methylsulfonyl)piperazin-1-yl)-3-(4-fluorophenylsulfonyl)quinoxaline 43

Yield: 60%; mp: 206–207°C. ^1^H NMR (500 MHz, CDCl_3_): δ 8.03 (d, 2H, *J = *8.5 Hz, aromatic H), 7.83 (d, 1H, *J = *8.0 Hz, aromatic H), 7.74 (t, 1H, *J = *7.0 Hz, aromatic H), 7.64 (d, 1H, *J = *8.0 Hz, aromatic H), 7.53 (t, 1H, *J = *7.5 Hz, aromatic H), 7.24 (d, 2H, *J = *8.0 Hz, aromatic H), 3.85 (t, 4H, *J = *4.5 Hz, piperazine H), 3.55 (t, 4H, *J = *4.5 Hz, piperazine H), 2.87 (s, 3H, CH_3_). ESI-MS (*m/z*): 451 [M+1]^+^.

#### General procedure for the synthesis of 44–48

Similar with procedure A, but *N*-carbamoylpiperazine was replaced by *N*-tosylpiperazine. Target compounds were obtained as yellow solid.

#### 2-(4-(4-Methylphenylsulfonyl)piperazin-1-yl)-3-(phenylsulfonyl)quinoxaline 44

Yield: 55%; mp: 161–163°C. ^1^H NMR (500 MHz, CDCl_3_): δ 7.90 (d, 2H, *J = *7.5 Hz, aromatic H), 7.80 (d, 1H, *J = *8.0 Hz, aromatic H), 7.72-7.68 (m, 3H, aromatic H), 7.65-7.60 (m, 2H, aromatic H), 7.50-7.46 (m, 3H, aromatic H), 7.37 (d, 2H, *J = *7.5 Hz, aromatic H), 3.78 (t, 4H, *J = *4.5 Hz, piperazine H), 3.31 (t, 4H, *J = *4.5 Hz, piperazine H), 2.45 (s, 3H, aromatic CH_3_). ESI-MS (*m/z*): 509 [M+1]^+^.

#### 2-(4-(4-Methylphenylsulfonyl)piperazin-1-yl)-3-(4-methylphenylsulfonyl)-quinoxaline 45

Yield: 43%; mp: 174–175°C. ^1^H NMR (500 MHz, CDCl_3_): δ 7.78-7.76 (m, 3H, aromatic H), 7.72-7.67 (m, 4H, aromatic H), 7.51 (t, 1H, *J = *7.5 Hz, aromatic H), 7.37 (d, 2H, *J = *7.5 Hz, aromatic H), 7.25 (d, 2H, *J = *7.5 Hz, aromatic H), 3.38 (t, 4H, *J = *4.5 Hz, piperazine H), 3.31 (t, 4H, *J = *4.5 Hz, piperazine H), 2.45 (s, 3H, aromatic CH_3_), 2.43 (s, 3H, aromatic CH_3_). ESI-MS (*m/z*): 523 [M+1]^+^.

#### 2-(4-(4-Methylphenylsulfonyl)piperazin-1-yl)-3-(4-methoxyphenylsulfonyl)-quinoxaline 46

Yield: 44%; mp: 172–175°C. ^1^H NMR (500 MHz, CDCl_3_): δ 7.84 (d, 2H, *J = *8.5 Hz, aromatic H), 7.79 (d, 1H, *J = *8.0 Hz, aromatic H), 7.72-7.66 (m, 4H, aromatic H), 7.50 (dt, 1H, *J = *8.5 and 1.5 Hz, aromatic H), 7.37 (d, 2H, *J = *8.5 Hz, aromatic H), 6.94 (dt, 2H, *J = *9.0 Hz, aromatic H), 3.87 (s, 3H, aromatic OCH_3_), 3.78 (t, 4H, *J = *4.5 Hz, piperazine H), 3.32 (t, 4H, *J = *4.5 Hz, piperazine H), 2.44 (s, 3H, aromatic CH_3_). ESI-MS (*m/z*): 539 [M+1]^+^.

#### 2-(4-(4-Methylphenylsulfonyl)piperazin-1-yl)-3-(4-bromophenylsulfonyl)-quinoxaline 47

Yield: 54%; mp: 214–216°C. ^1^H NMR (500 MHz, CDCl_3_): δ 7.80 (d, 1H, *J = *7.5 Hz, aromatic H), 7.77 (d, 2H, *J = *9.0 Hz, aromatic H), 7.72-7.70 (m, 3H, aromatic H), 7.66 (d, 1H, *J = *8.0 Hz, aromatic H), 7.63 (d, 2H, *J = *9.0 Hz, aromatic H), 7.52 (dt, 1H, *J = *8.5 and 1.5 Hz, aromatic H), 7.37 (d, 2H, *J = *8.5 Hz, aromatic H), 3.79 (t, 4H, *J = *4.5 Hz, piperazine H), 3.31 (t, 4H, *J = *4.5 Hz, piperazine H), 2.45 (s, 3H, aromatic CH_3_). ESI-MS (*m/z*): 589 [M+1]^+^.

#### 2-(4-(4-Methylphenylsulfonyl)piperazin-1-yl)-3-(4-fluorophenylsulfonyl)-quinoxaline 48

Yield: 86%; mp: 172–174°C. ^1^H NMR (500 MHz, CDCl_3_): δ 7.94 (dd, 2H, *J = *7.0 and 5.0 Hz, aromatic H), 7.80 (d, 1H, *J = *8.0 Hz, aromatic H), 7.71-7.70 (m, 3H, aromatic H), 7.64 (d, 1H, *J = *7.5 Hz, aromatic H), 7.51 (dt, 1H, *J = *7.5 and 1.0 Hz, aromatic H), 7.37 (d, 2H, *J = *8.5 Hz, aromatic H), 7.18 (t, 2H, *J = *8.5 Hz, aromatic H), 3.80 (t, 4H, *J = *4.5 Hz, piperazine H), 3.32 (t, 4H, *J = *4.5 Hz, piperazine H), 2.45 (s, 3H, aromatic CH_3_). ESI-MS (*m/z*): 527 [M+1]^+^.

#### General procedure for the synthesis of 49–53

Similar with procedure A, but *N*-carbamoylpiperazine was replaced by 3-(morpholinopropyl)piperazine. Target compounds were obtained as yellow mucous substance.

#### 2-(4-(3-Morpholinopropyl)piperazin-1-yl)-3-(phenylsulfonyl)quinoxaline 49

Yield: 57%; ^1^H NMR (500 MHz, CDCl_3_): δ 8.00 (d, 2H, *J = *7.0 Hz, aromatic H), 7.77 (d, 1H, *J = *8.5 Hz, aromatic H), 7.68 (dt, 1H, *J = *8.0 and 1.0 Hz, aromatic H), 7.68 (t, 2H, *J = *7.5 Hz, aromatic H), 7.54 (t, 2H, *J = *8.0 Hz, aromatic H), 7.45 (dt, 1H, *J = *8.5 and 1.0 Hz, aromatic H), 3.81 (m, 4H, piperazine H), 3.75 (t, 4H, *J = *4.5 Hz, morpohline H), 2.79 (t, 4H, *J = *4.5 Hz, morpholine), 2.56 (t, 2H, *J = *7.5 Hz, CH_2_), 2.49 (m, 4H, piperazine H), 2.45 (t, 2H, *J = *7.5 Hz, CH_2_), 1.82-1.78 (m, 2H, CH_2_). ESI-MS (*m/z*): 482 [M+1]^+^.

#### 2-(4-(3-Morpholinopropyl)piperazin-1-yl)-3-(4-methylphenylsulfonyl)quinoxaline 50

Yield: 58%; ^1^H NMR (500 MHz, CDCl_3_): δ 7.88 (d, 2H, *J = *8.0 Hz, aromatic H), 7.78 (d, 1H, *J = *8.5 Hz, aromatic H), 7.69-7.65 (m, 2H, aromatic H), 7.48 (dt, 1H, *J = *8.0 and 1.0 Hz, aromatic H), 7.34 (d, 2H, *J = *8.0 Hz, aromatic H), 3.90-3.88 (m, 4H, piperazine H), 3.78 (t, 4H, *J = *4.5 Hz, morpohline H), 2.96 (m, 4H, piperazine H), 2.72 (t, 2H, *J = *7.0 Hz, CH_2_), 2.55-2.50 (m, 6H, morpholine H and CH_2_), 2.45 (s, 3H, aromatic CH_3_),1.96-1.93 (m, 2H, CH_2_). ESI-MS (*m/z*): 497 [M+1]^+^.

#### 2-(4-(3-Morpholinopropyl)piperazin-1-yl)-3-(4-methoxyphenylsulfonyl)-quinoxaline 51

Yield: 64%; ^1^H NMR (500 MHz, CDCl_3_): δ 7.94 (d, 2H, *J = *9.0 Hz, aromatic H), 7.77 (d, 1H, *J = *8.5 Hz, aromatic H), 7.67-7.64 (m, 2H, aromatic H), 7.46 (t, 1H, *J = *8.0 Hz, aromatic H), 7.00 (d, 2H, *J = *8.5 Hz, aromatic H), 3.88 (s, 3H, aromatic OCH_3_), 3.82-3.79 (m, 4H, piperazine H), 3.75 (t, 4H, *J = *4.5 Hz, morpohline H), 2.83 (m, 4H, piperazine H), 2.59 (t, 2H, *J = *8.0 Hz, CH_2_), 2.50 (t, 4H, *J = *4.5 Hz, morpholine H), 2.47 (t, 2H, *J = *8.0 Hz, CH_2_), 1.85-1.79 (m, 2H, CH_2_). ESI-MS (*m/z*): 512 [M+1]^+^.

#### 2-(4-(3-Morpholinopropyl)piperazin-1-yl)-3-(4-bromophenylsulfonyl)quinoxaline 52

Yield: 78%; ^1^H NMR (500 MHz, CDCl_3_): δ 7.86 (d, 2H, *J = *9.0 Hz, aromatic H), 7.77 (d, 1H, *J = *8.0 Hz, aromatic H), 7.69-7.66 (m, 3H, aromatic H), 7.68 (d, 1H, *J = *8.5 Hz, aromatic H), 7.46 (dt, 1H, *J = *8.0 and 1.5 Hz, aromatic H), 3.82 (t, 4H, *J = *4.5 Hz, piperazine H), 3.75 (t, 4H, *J = *4.5 Hz, morpohline H), 2.76 (t, 4H, *J = *4.5 Hz, piperazine H), 2.54 (t, 2H, *J = *7.5 Hz, CH_2_), 2.48 (t, 4H, *J = *4.5 Hz, morpholine H), 2.44 (t, 2H, *J = *7.5 Hz, CH_2_), 1.82-1.76 (m, 2H, CH_2_). ESI-MS (*m/z*): 562 [M+1]^+^.

#### 2-(4-(3-Morpholinopropyl)piperazin-1-yl)-3-(4-fluorophenylsulfonyl)quinoxaline 53

Yield: 75%; ^1^H NMR (500 MHz, CDCl_3_): δ 8.02-7.99 (m, 2H, aromatic H), 7.76 (d, 1H, *J = *8.5 Hz, aromatic H), 7.68 (t, 1H, *J = *7.5 Hz, aromatic H), 7.61 (d, 1H, *J = *8.5 Hz, aromatic H), 7.45 (t, 1H, *J = *8.0 Hz, aromatic H), 7.22 (t, 2H, *J = *8.5 Hz, aromatic H), 3.82 (m, 4H, piperazine H), 3.74 (t, 4H, *J = *4.5 Hz, morpohline H), 2.80 (t, 4H, *J = *4.5 Hz, morpholine), 2.57 (t, 2H, *J = *7.5 Hz, CH_2_), 2.49 (m, 4H, piperazine H), 2.46 (t, 2H, *J = *7.5 Hz, CH_2_), 1.84-1.78 (m, 2H, CH_2_). ESI-MS (*m/z*): 500 [M+1]^+^.

### MTT Assay - Inhibition of Human Cancer Cell Lines

Human prostate cancer PC3 cells, lung adenocarcinoma epithelial A549 cells, colon cancer HCT116 cells, promyelocytic leukemia HL60 cells and nasopharyngeal carcinoma KB cells were obtained from the cell bank of the Institute of Biochemistry and Cell Biology, Chinese Academy of Sciences (Shanghai, China). The inhibitory activity of target compounds against tested cancer cell lines was measured using the MTT assay. PC3, A549, HCT116, HL60, and KB cancer cell lines were cultured in RPMI-1640 medium (Invitrogen, Carlsbad, CA, USA) with heat-inactivated 10% fetal bovine serum, penicillin (100 units/mL) and streptomycin (100 µg/mL) and incubated in an atmosphere with 20% oxygen and 5% carbon dioxide at 37°C. All tested compounds were dissolved in DMSO at concentrations of 10.0 mg/mL and diluted to appropriate concentrations. Cells were plated in 96-well plates for 24 h and subsequently treated with different concentrations of all tested compounds for 72 h. Viable cells were determined using 3-(4,5-dimethylthiazol-2-yl)-2,5-diphenyltetrazolium bromide assay kit (MTT, Sigma) according to operation instructions provided by the manufacturer. The concentration of drug causing 50% inhibition in absorbance compared with control cells (IC_50_) was calculated using the software of dose-effect analysis with microcomputers.

### FP Assay - Inhibition of PI3Kα

The PI3Kα inhibitory test was determined using a competitive fluorescence polarization kinase activity assay. PI3K fluorescence polarization assay kit (catalog No. K-1100) and recombinant human PI3Kα (catalog No. E-2000) were purchased from Echelon Biosciences (Salt Lake City, UT, USA). PI3K reactions were performed in 5 mM HEPES, Ph 7, 2.5 mM MgCl_2_, 10 mM DTT and 50 µM ATP, using diC_8_-phosphatidylinositol-4, 5-bisphosphate (PIP_2_) as the substrate, and the final reaction volumes were 10 µL. For evaluation of the PI3Kα inhibitory activity of target compounds, 50 ng enzyme and 10 µM substate were used per 10 µL reaction volume with the concentrations of inhibitors ranging from 3.2 nM to 10 µM. After incubating for 3 h at room temperature, reactions were quenched by adding chelators. A mixture of phosphoinositide binding protein was added and mixed, followed by the addition of a fluorophore-labeled phosphoinositide tracer. Samples were then mixed in 384-well black Corning nonbinding plates (Corning, NY, USA) and incubated in a dark environment for 1 h to equilibrate. Finally, polarization values were measure using red fluorophores with appropriate filters to determine the extent of enzyme activity in the reaction.

### Flow Cytometry Analysis

For flow cytometry analysis, PC3 cells were treated with DMSO, compound **41** and GDC0941 for 24 h. Cells were washed twice with PBS and fixed in 75% ethanol at −20°C. The cell pellet was resuspended in 100 µL of PBS containing 200 mg/mL RNase (Amerson, Solon, OH, USA), then incubated at 37°C for 0.5 h. After incubation, the cells were stained with 20 mL/L propidium iodide (PI, Sigma, St. Louis, MO, USA) at 4°C for 15 min. The fluorescence of cell was measured with FACSCalibur flow cytometer (Becton-Dickinson, Lincoln Park, NJ, USA).

### Molecular Docking

The X-ray co-crystal structure of mutant PI3Kα-wortmannin complex was downloaded from the RCSB Protein Data Bank (ID: 3HHM). The C-Dock protocol within DiscoveryStudio 2.1 was utilized to perform molecular docking analysis for compounds **22** and **41**. For ligand preparation, the 3D structures of **22** and **41** were generated and minimized using DiscoveryStudio 2.1. For protein preparation, the hydrogen atoms were added, water was removed, and the CHARMm force field was employed. The whole PI3Kα enzyme was defined as a receptor and the site sphere was selected based on the ligand binding location of wortmannin. Compound **22** or **41** were placed in the binding site during the docking procedure. Docking parameters were set as follows: top hits, 25; random conformations, 25; random conformations dynamics steps, 1000; grid extension, 8.0; random dynamics time step, 0.002. All other parameters were set as default values. Types of interactions of the docked enzyme with ligand were analyzed upon the finish of molecular docking. All graphical pictures were made using PyMol.
